# Operative Surgery

**Published:** 1887-03

**Authors:** 


Manual of Operative Surgery. By Joseph D. Bryant, M. D. D. Appleton & Company, New York. 1887.

The first of the above named works contains 875 octavo pages with 1,005 illustrations, and is a carefully revised and greatly improved edition of the hand-book of surgery, which has been long and favorably known to the profession. It is now a most complete guide for the performance of the recognized operations in the practice of surgery, and is as concise as is compatible in the description of the various processes and details required in their execution.

The second on the list contains 530 octavo pages, and has 793 illustrations, being a great improvement upon the production of the same author, which appeared as a part of the Birmingham Library some years ago.

If any comparison of these two works on operative surgery may be based upon their respective directions for proceeding in cutting for strangulated hernia, it will be observed that Smith is more definite and precise in his instructions, while Bryant impresses the operator with the great caution requisite to avoid injury to the contiguous arteries without indicating the exact mode in which this great *desideratum* is to be accomplished. In this respect he does not follow in the lead of his British prototype and namesake, who defines the incisions with clearness that are requisite in relieving the stricture in strangulated inguinal and femoral hernias.

Our space does not permit such a notice of the valuable contents of these books as the high standing of the authors would warrant, but with little that is objectionable in either, much is found for commendation in both, and with the lesser work of Stimson before the public, the practical discernment of our progressive age will not fail in making a selection suited to the sphere of the individual operator.

The elegant volume in sheep from the house of Lea Brothers & Co., and the chaste and artistic cloth binding of D. Appleton & Co., with the printing and illustrations of the highest order of excellence in both of these books, reflect credit upon their respective publishers.

